# Antimicrobial Efficacy of Intracanal Medicaments Incorporated With Nanoparticles in Primary Teeth: An In Vitro Study

**DOI:** 10.1155/tswj/5182716

**Published:** 2025-06-09

**Authors:** Azra Kaukab, Sridhar Nekkanti

**Affiliations:** Department of Pediatric and Preventive Dentistry, Manipal College of Dental Sciences, Manipal Academy of Higher Education, Manipal, India

## Abstract

Placing an intracanal medicament helps in the disinfection of primary root canals, and nanoparticles enhance the material properties by increasing the physical and chemical reactivity. The study was aimed at assessing the efficacy of calcium hydroxide+calcium hydroxide nanoparticles and zinc oxide+zinc oxide nanoparticles as an intracanal medicament when compared with calcium hydroxide and zinc oxide eugenol in terms of antibacterial efficacy and penetration depth. Forty extracted human primary teeth were included in the study. Of these, 40 teeth were divided into two subgroups—Group A (to assess antibacterial efficacy) and Group B (to assess penetration depth). The teeth in Group A were contaminated with an *Enterococcus faecalis* biofilm for 7 days, and CFU count was determined. Calcium hydroxide (A1), calcium hydroxide with calcium hydroxide nanoparticles (A2), zinc oxide with zinc oxide nanoparticles (A3), and zinc oxide eugenol (A4) were placed in the canals. CFU count was determined on Day 7 and Day 14. The teeth in Group B were also divided into calcium hydroxide (B1), calcium hydroxide with calcium hydroxide nanoparticles (B2), zinc oxide with zinc oxide nanoparticles (B3), and zinc oxide eugenol (B4) groups. Two teeth from each group were subjected to SEM analysis. A statistically significant difference was noted in the CFU count reduction on Day 7 in Group A2, Group A3, and Group A4. The highest penetration depth was noted in Group B2. The authors concluded that the antibacterial efficacy and penetration depth into dentin improved on incorporating calcium hydroxide and zinc oxide nanoparticles. Based on this conclusion, the authors recommend further in vivo studies with nanoparticles incorporated intracanal medicaments in primary teeth to determine their safety of use and feasibility in a clinical scenario.

## 1. Introduction

Pulpectomy is an endodontic procedure for irreversibly inflamed or necrotic pulp tissue due to caries or trauma [1]. Due to the anatomic complexities and physiological resorption in primary root canals, complete mechanical debridement is difficult. Following debridement of the pulp tissue and copious irrigation, placing an intracanal medicament helps in the disinfection of the root canal to receive a biologically acceptable obturating material. Placing an intracanal medicament helps in preventing bacterial regrowth and improves bacterial suppression, successfully eliminating bacterial flora. Higher penetration depth allows the medicament particles to reach deep into the dentinal tubules where the bacteria persist. Owing to the antibacterial property of the medicament particles, interaction with the microbial cells leads to cell death and elimination of infection [2, 3].

Nanotechnology has been used extensively in dentistry to improve material properties. They have varying dimensions in size ranging from 1 to 100 nm. Due to their ultrasmall sizes, they have a higher surface area to mass ratio, leading to increased physical and chemical reactivity. They have shown improved antibacterial action because of the high charge density that causes an interaction with the negatively charged cell wall and subsequently the cell membrane of the bacteria. Protein synthesis is inhibited, which eventually prevents replication of DNA, causing cell death. Another mechanism involved in cell lysis is the release of reactive nitric oxide species [4].

Calcium hydroxide gained popularity as an intracanal medicament due to its broad antimicrobial spectrum, alkalizing effect, and inflammatory control. If calcium hydroxide nanoparticles are incorporated in the medicament, there could be a potential benefit of better penetration into the dentinal tubules and hence increased antibacterial efficacy [3, 4].

Zinc oxide is usually used with eugenol as an obturation material in primary teeth. But due to its antibacterial properties, it can also be used as an intracanal medicament. Its antibacterial activity can be enhanced by using it in its nanoparticle form [5, 6].

There is a scarcity of literature regarding the effective use of nanoparticles in primary teeth as an intracanal medicament.

*Enterococcus faecalis* is a facultative anaerobic gram-positive microorganism that is found in the root canal. It has high resistance to antimicrobials due to various virulence factors. Studies have shown that *E. faecalis* is the primary organism that causes root canal failure; this is due to the incomplete elimination of *E. faecalis* from the root canal. It has also been demonstrated that *E. faecalis* can penetrate deep into the dentinal tubules and escape interaction with intracanal medicaments. Hence, *E. faecalis* is the most important organism to be assessed while evaluating the antibacterial activity of any intracanal medicament [7].

Microbes that persist in dentinal tubules of the root, even after biomechanical preparation, cause endodontic treatment failure. This is because they recolonize the dentinal tubules and cause persistent infection. The chances of endodontic treatment failure can be reduced by placing an intracanal medicament in the canal to eliminate the bacterial biofilm from the dentinal tubules in the root [8].

*E. faecalis* is highly resistant to the antibacterial mechanisms of conventionally used intracanal medicaments like calcium hydroxide [9]. Thus, there have been advancements in the composition of intracanal medicaments like using more potent agents like chlorhexidine gluconate, curcumin, and triple antibiotic paste (metronidazole, ciprofloxacin, and minocycline) [10].

One method that has recently been introduced is incorporating nanoparticles in the intracanal medicament. Their smaller size helps in achieving a better penetration depth into the dentinal tubule, and the increased surface charge density increases its antibacterial properties [4].

As infected root canals usually have multispecies biofilm, previous studies have shown triple antibiotic paste (metronidazole, ciprofloxacin, and minocycline) to be highly effective in the disinfection process. However, triple antibiotic paste can cause the discoloration of the tooth (attributed to minocycline) and the emergence of resistant bacteria [8]. The use of nanoparticles can be advantageous in this scenario, particularly functionalized nanoparticles, as these can specifically eliminate bacteria without damaging the host cells [11].

Hence, this study was designed to evaluate the penetration depth and antibacterial efficacy of calcium hydroxide and zinc oxide nanoparticles when used in primary teeth.

A good intracanal medicament is of prime importance particularly in primary teeth. However, most studies have established the effectiveness of nanoparticle incorporated intracanal medicaments in permanent teeth [5, 6, 12]. There is a scarcity of literature regarding the effective use of nanoparticles in primary teeth as an intracanal medicament [4, 13].

The objective of the study was to assess the antibacterial efficacy of calcium hydroxide+calcium hydroxide nanoparticles and zinc oxide+zinc oxide nanoparticles as intracanal medicaments when compared with calcium hydroxide and zinc oxide eugenol by assessing the reduction in colony forming units and penetration depth into dentinal tubules.

## 2. Materials and Methods

Sample size calculation: It was calculated using the G power 3.1.9.4 software. With the power of the study at 80%, effect size of 0.5, and mean difference of 0.6 [14], the total sample size was calculated to be 36. This was rounded off to 40. To confirm the presence of *E. faecalis* biofilm, three teeth were added. These were sectioned longitudinally and subjected to SEM (scanning electron microscope) analysis. The final sample size included 43 teeth [14].

Inclusion and exclusion criteria: Human primary teeth extracted due to physiologic or gross carious destruction with at least two-thirds of radicular length were included in the study. Teeth with pathologic resorption, excessive curvature in the root, surface perforations, and calcification of root canals were excluded.

Tooth preparation: The teeth were picked based on the aforementioned criteria, following which they were placed in saline solution (sodium chloride injection IP 0.9%x/v, Fresenius Kabi India Pvt. Ltd., Pune, India) and subsequently soaked in 5% sodium hypochlorite (Medilise Chemicals, Kannur, Kerala, India) to eliminate soft tissue remains from the root surface. The tooth crown was sliced using a low-speed diamond disk (NMD Nexus Medodent, Mumbai, Maharashtra). Each root canal was then instrumented with #15–#35 K file. Profuse irrigation with 1% sodium hypochlorite, 17% EDTA (17% ED, Dental Products of India, The Bombay Burmah Trading Corporation Ltd., Rudrapur, Uttarakhand, India), and saline was done between increasing file sizes (#15–35, Mani Inc., Tochigi, Japan). The canals were then dried using paper points (2% #35, DiaDent Group International, Burnaby, Canada). The prepared teeth were sealed in sterile pouches and autoclaved.

Nanoparticle synthesis and characterization: Zinc oxide and calcium hydroxide nanoparticles were synthesized chemically (Manufactured at White Lab, Material Research Centre, Saveetha Dental College, Chennai, India). X-ray diffraction was used to characterize the nanoparticles.

Randomization and allocation: Forty teeth were allocated into two groups using simple randomized sampling with a random table of numbers:

• Group A (*n* = 20, determination of antibacterial efficacy)
• Group B (*n* = 20, determination of penetration depth)

Microbial film contamination: The 20 teeth in Group A were contaminated with an *E. faecalis* biofilm (American Type Culture Collection 29212). The three additional teeth were also contaminated with the microbial film along with the teeth in the group.

Incubation was carried out for 7 days at 37°C. After this, the three samples were sectioned and viewed under a SEM (Zeiss Evo-10, Carl-Zeiss, Oberkochen, Germany) to confirm the presence of the biofilm. Once the biofilm presence was confirmed, the three tooth samples were discarded.

A swab was then taken using sterile paper points in four teeth randomly to determine the CFU count before intracanal medicament placement.

Determination of antimicrobial efficacy: The 20 teeth were further randomly allocated into four subgroups based on the intracanal medicament placed:

Group A:

• Subgroup A1 (*n* = 5): calcium hydroxide (Raman Research, Kolakata, India)
• Subgroup A2 (*n* = 5): calcium hydroxide with calcium hydroxide nanoparticles
• Subgroup A3 (*n* = 5): zinc oxide eugenol (Zinc Oxide, Dental Products of India, The Bombay Burmah Trading Corporation Ltd., Rudrapur, Uttarakhand, India; Eugenol, Dental Products of India, The Bombay Burmah Trading Corporation Ltd., Rudrapur, Uttarakhand, India)
• Subgroup A4 (*n* = 5): zinc oxide with zinc oxide nanoparticles

Propylene glycol was used as the vehicle in Subgroups A1, A2, and A4. It was mixed with the material until a creamy consistency was obtained, and a lentulospiral (#25, Mani INC., Tochigi, Japan) at low speed was used to place the material in the prepared root canal. Incubation at 37°C was continued for 14 days. Dentinal shavings were taken from one tooth in each group on Day 7 and Day 14. The CFU count was determined from these dentinal shavings.

Determination of particle density and penetration depth: The teeth in Group B were further divided into four subgroups based on the intracanal medicament placed:

Group B:

• Subgroup B1 (*n* = 5): calcium hydroxide
• Subgroup B2 (*n* = 5): calcium hydroxide with calcium hydroxide nanoparticles
• Subgroup B3 (*n* = 5): zinc oxide eugenol
• Subgroup B4 (*n* = 5): zinc oxide with zinc oxide nanoparticles

Propylene glycol (Ases Chemical Works, Jodhpur, Rajasthan) was used in Subgroups B1, B2, and B4 as a vehicle to carry the medicament into the canal. It was mixed with the material until a creamy consistency was obtained, and a lentulospiral at low speed was used to place the material in the prepared root canal. The samples were incubated at 37°C for 7 days. Then, the samples were sectioned longitudinally in the coronal to apical direction using a double-sided diamond disk with mandrel (NMD Nexus Medodent, Mumbai, Maharashtra) mounted on a micromotor. The samples were then subjected to examination under the SEM (Zeiss Evo-10, Carl-Zeiss, Oberkochen, Germany) at 3.00 K X magnification to evaluate penetration depth. The tooth section was also examined in the cervical, middle, and apical thirds of the root to determine the density of nanoparticles. The density of the nanoparticles was quantified by counting the number of particles in a 3 *μ*m^2^ area in a 3.00 K X magnified image.

## 3. Results

Statistical package SPSS 26.0 (SPSS Inc., Chicago, IL) was used to analyze data with the level of significance set at *p* < 0.05. Descriptive statistics were performed to assess the mean and standard deviation of the groups. The Shapiro–Wilkinson test was used to determine the normality of the data. Inferential statistics was used to evaluate the difference between the groups. It was done using one-way ANOVA (three groups) followed by Bonferroni's post hoc test. Intragroup analysis was done using repeated measures of ANOVA followed by Bonferroni's post hoc test. One-way ANOVA was carried out to compare CFU per milliliter between the groups and within each group on Day 0, Day 7, and Day 14.

The colony forming units seen in all the groups of Day 0, Day 7, and Day 14 are depicted in Table 1. From this, intergroup and intragroup comparisons were carried out (Table 2). Intragroup analysis on Day 7 showed significant reductions in groups A2(Ca(OH)_2_+Ca(OH)_2_ NP), A3(ZnOE), and A4(ZnO+ZnO NP) when compared to Group A1 (Ca(OH)_2_) (Table 2).

**Figure 1:**
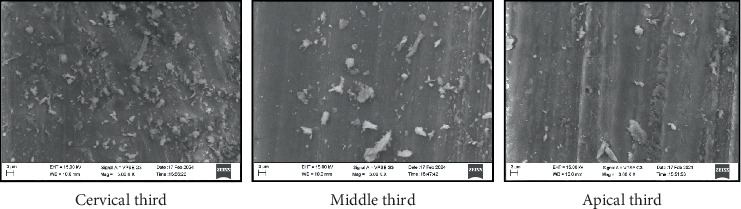
SEM image depicting particle density—Group B1: calcium hydroxide.

**Figure 2:**
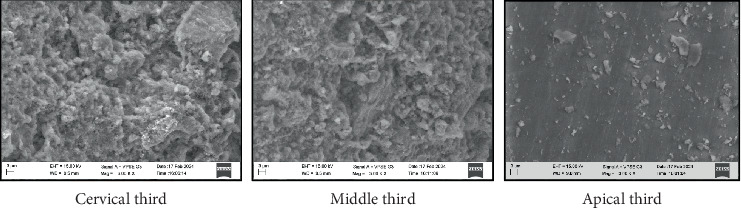
SEM image depicting particle density—Group B2: calcium hydroxide with calcium hydroxide nanoparticles.

**Figure 3:**
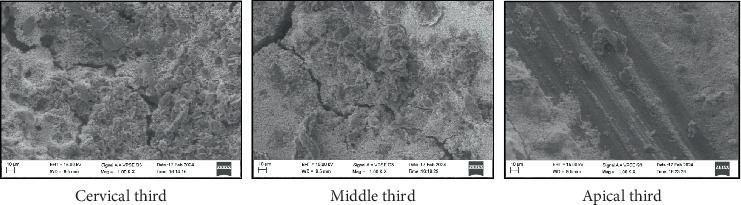
SEM image depicting particle density—Group B3: zinc oxide eugenol.

**Figure 4:**
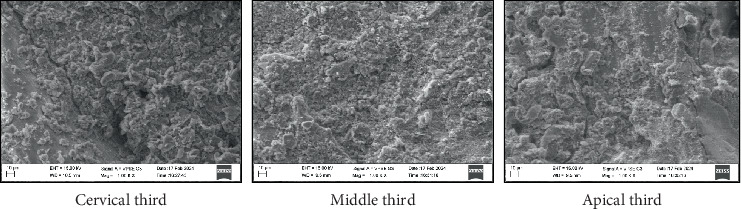
SEM image depicting particle density—Group B4: zinc oxide with zinc oxide nanoparticles.

All four groups showed maximum particle density in the cervical third compared to the middle and apical third of the root canal (Figures 1, 2, 3, and 4) (Table 3). Maximum penetration depth was noted in Group B2 (Ca(OH)_2_+Ca(OH)_2_ NP) when compared with the other three groups (Table 4).

## 4. Discussion

The results of this study found the *E. faecalis* CFU counts to be significantly lower in the nanoparticle groups (Group B1 and Group C1) on Day 7. Similar findings were observed in the in vitro study done by Moradi and Haghgoo (2018). The authors assessed the antibacterial efficacy of a nanosilver solution, sodium hypochlorite, and normal saline solution when used for root canal irrigation and found a significant reduction in the number of colony forming unit counts of the nanosilver and sodium hypochlorite groups when compared to saline, indicating that the nanosilver solution can be used effectively for irrigation [15].

The findings of this study are also in accordance with Dianat et al., Balto et al., and Afkhami et al., wherein adding nanoparticles to calcium hydroxide increased its antibacterial efficacy against *E. faecalis* [9, 16, 17].

Moheb et al. compared the effect of five intracanal medicaments on *E. faecalis* biofilm inside the root canal. They assessed the antibacterial efficacy of calcium hydroxide alone, calcium hydroxide in combination with chlorhexidine, metronidazole alone, metronidazole in combination with chlorhexidine, chitosan in combination with chlorhexidine, and chitosan alone. Microbial CFU counts in all the groups after placement of the intracanal medicament for 2 weeks reduced significantly [18]. This finding is in accordance with our study, where placement of the intracanal medicament resulted in a reduction of CFU counts in all four groups. Another study by Tandon et al. also reported a significant reduction in the *E. faecalis* counts on placing calcium hydroxide and chlorhexidine as intracanal medicaments for 7 days [19].

Conferring with the findings of this study, Teja et al. also reported a higher reduction in *E. faecalis* counts when calcium hydroxide was placed in combination with silver nanoparticles as opposed to the placement of calcium hydroxide alone as the intracanal medicament [20].

Guerreiro-Tanomaru et al. reported that the incorporation of calcium hydroxide and zinc oxide micro/nanoparticles had no influence on the antimicrobial activity [14]. This study contradicts these findings because intracanal medicament incorporated with zinc oxide and calcium hydroxide nanoparticles showed increased antibacterial activity when compared to conventional calcium hydroxide.

Highest amount of reduction was seen in Group D1, where zinc oxide eugenol was used as the intracanal medicament. This can be attributed to the antibacterial properties of eugenol [21].

A lentulospiral was used to place the intracanal medicament in all the groups to allow uniform distribution of the medicament with lesser voids [22]. As this was done by mounting the lentulospiral on a slow-speed handpiece in all the samples, it eliminated the possibility of variation that is involved in placing the medicament manually using a finger/hand spreader [22].

This study showed a higher area of distribution of the medicament in the cervical/middle areas of the canal when compared to the apical third, which is in line with the observations of Thonai et al. who found the dentinal penetration depth and density to be significantly higher in the cervical third of the root when compared to the apical/middle third. This could probably be due to the presence of debris and the smear layer, which can be attributed to the ineffectiveness of mechanical debridement and irrigation in the apical third due to the narrow canal diameter in this region [23].

The SEM images showed the highest penetration of the medicament particles in Group B2 (calcium hydroxide with calcium hydroxide nanoparticles) followed by Group C2 (zinc oxide with zinc oxide nanoparticles). This is due to the smaller and uniform size of the nanoparticles that were incorporated in these groups [24]. This finding agrees with Zand et al. and Farzaneh et al. who also found significantly higher penetration depth into the dentinal tubules with calcium hydroxide nanoparticles when compared to conventional calcium hydroxide [3, 24]. Prashanth et al. also reported significantly higher penetration depth in the nanocalcium hydroxide, nanocurcumin, and nanochitosan groups when compared to the conventional calcium hydroxide, curcumin, and chitosan, which are in accordance with the results of this study [13].

In contrast to this study, Thonai et al. [23] found a higher penetration depth into the dentinal tubules when calcium hydroxide was combined with 2% chlorhexidine gluconate and chitosan as opposed to calcium hydroxide nanoparticles. Among the two, the combination of calcium hydroxide with chlorhexidine showed the highest penetration depth. They attributed this to the strong cationic action of chlorhexidine causing a strong affinity to the phosphate anions present in dentin. Due to this electrostatic interaction, chlorhexidine has a high adsorption power [22].

Çalt and Serper showed that calcium hydroxide particles do not permeate the dentinal tubules and remain on the surface [25]. This study also showed similar findings in Groups B (Figure 1) (calcium hydroxide) and B4 (Figure 4) (zinc oxide eugenol), where the particles were seen clogged on the dentinal surface without penetrating them. The particles were also more irregularly distributed and less densely packed.

The limitations of this study include the use of a smaller sample size, which affects the generalizability of the results. Also, only a 1-week-old *E. faecalis* biofilm was used, and the antimicrobial resistance of which may not be comparable to the several month-old biofilms found in the root canals of teeth present in the oral cavity. Du et al. and Birring et al. showed that bacteria in young biofilms are vulnerable to the action of irrigants and intracanal medicaments when compared to older, more mature biofilms [26, 27]. In addition, the results of this study are difficult to extrapolate to clinical situations because root canals in the oral cavity have microbially diverse biofilms that are more difficult to eliminate.

## 5. Conclusion

The authors concluded that the antibacterial efficacy and penetration depth into dentin improved on incorporating calcium hydroxide nanoparticles with calcium hydroxide and zinc oxide nanoparticles with zinc oxide when compared to calcium hydroxide. Zinc oxide eugenol showed the highest antibacterial efficacy compared to all the other groups. Zinc oxide and calcium hydroxide nanoparticles can be added to intracanal medicaments to enhance antibacterial efficacy and penetration depth into dentinal tubules. Based on this conclusion, the authors recommend further in vivo studies with nanoparticles incorporated into intracanal medicaments in primary teeth to determine their safety of use and feasibility in a clinical scenario.

## Figures and Tables

**Table 1:** Number of colony forming units seen in each group on Day 0, Day 7, and Day 14.

**Groups**	**Day 0**	**Day 7 (** **m** **e** **a** **n** ± **SD)**	**Day 14**
Group A1 Calcium hydroxide	10^8^	(0.12 ± 0.02) × 10^2^	No growth
Group A2 Calcium hydroxide+calcium hydroxide NP	10^8^	(0.0065 ± 0.0035) × 10^2^	No growth
Group A3 Zinc oxide eugenol	10^8^	(0.015 ± 0.005) × 10^2^	No growth
Group A4 Zinc oxide+zinc oxide nanoparticles	10^8^	No growth	No growth

**Table 2:** Intergroup and intragroup comparison of CFU per milliliter on Day 0, Day 7, and Day 14.

		**Sum of squares**	**Degrees of freedom**	**Mean square**	**F**	**Sig.**
Day 0	Between groups	0.000	3	0.000		
	Within groups	0.000	16	0.000		
	Total	0.000	19			

Day 7	Between groups	4.658	3	1.533	53.745	0.0001⁣^∗^
	Within groups	0.462	16	0.029		
	Total	5.120	19			

Day 14	Between groups	0.000	3	0.000		
	Within groups	0.000	16	0.000		
	Total	0.000	19			

*Note:* One-way ANOVA.

∗*p* < 0.05 is considered statistically significant (*p* > 0.05 as per Shapiro–Wilkinson test).

**Table 3:** Density of intracanal medicament particles (ICM/3 *μ*m^2^ area) (mean ± SD value).

**No. of particles/groups**	**Cervical third**	**Middle third**	**Apical third**
Group B1 Calcium hydroxide	3 ± 0.10	1 ± 0.05	1 ± 0.05
Group B2 Calcium hydroxide+calcium hydroxide NP	6 ± 0.25	6 ± 0.25	3 ± 0.25
Group B3 Zinc oxide eugenol	2 ± 0.25	1 ± 0.25	1 ± 0.05
Group B4 Zinc oxide+zinc oxide NP	8 ± 0.10	7 ± 0.50	4 ± 0.25

**Table 4:** Penetration depth of intracanal medicament particles into dentinal tubules (mean ± SD value).

**Groups**	**Penetration depth**
Group B1 Calcium hydroxide	18 ± 0.5* μ*m
Group B2 Calcium hydroxide+calcium hydroxide NP	42 ± 1* μ*m
Group B3 Zinc oxide eugenol	16.6 ± 0.5* μ*m
Group B4 Zinc oxide+zinc oxide NP	36 ± 0.5* μ*m

## Data Availability

All the data used for analysis in the study are available with the corresponding author and can be produced upon request.
